# Relationships Linking Amplification Level to Gene Over-Expression in Gliomas

**DOI:** 10.1371/journal.pone.0014249

**Published:** 2010-12-08

**Authors:** Nicolas Vogt, Anne Gibaud, Anna Almeida, Isabelle Ourliac-Garnier, Michelle Debatisse, Bernard Malfoy

**Affiliations:** 1 Institut Curie, Centre de Recherche, Paris, France; 2 CNRS UMR3244, Paris, France; 3 UPMC, Paris, France; Institute of Cancer Research, United Kingdom

## Abstract

**Background:**

Gene amplification is thought to promote over-expression of genes favouring tumour development. Because amplified regions are usually megabase-long, amplification often concerns numerous syntenic or non-syntenic genes, among which only a subset is over-expressed. The rationale for these differences remains poorly understood.

**Methodology/Principal Finding:**

To address this question, we used quantitative RT-PCR to determine the expression level of a series of co-amplified genes in five xenografted and one fresh human gliomas. These gliomas were chosen because we have previously characterised in detail the genetic content of their amplicons. In all the cases, the amplified sequences lie on extra-chromosomal DNA molecules, as commonly observed in gliomas. We show here that genes transcribed in non-amplified gliomas are over-expressed when amplified, roughly in proportion to their copy number, while non-expressed genes remain inactive. When specific antibodies were available, we also compared protein expression in amplified and non-amplified tumours. We found that protein accumulation barely correlates with the level of mRNA expression in some of these tumours.

**Conclusions/Significance:**

Here we show that the tissue-specific pattern of gene expression is maintained upon amplification in gliomas. Our study relies on a single type of tumour and a limited number of cases. However, it strongly suggests that, even when amplified, genes that are normally silent in a given cell type play no role in tumour progression. The loose relationships between mRNA level and protein accumulation and/or activity indicate that translational or post-translational events play a key role in fine-tuning the final outcome of amplification in gliomas.

## Introduction

The development of tumours often relies on genomic rearrangements that alter the expression of genes favouring growth and survival pathways. Among rearrangements frequently involved in tumour progression is DNA amplification, which drastically modifies gene dosage in cancer cells [1], [2]. Amplified sequences may be found within the chromosomes, clustered within homogeneously staining regions (HSRs) or spread among different loci. Alternatively, they may lie on circular extra-chromosomal DNA molecules called double minutes (dmins) [3], [4]. Regardless of the localisation of extra copies, amplification events often lead to the co-amplification of groups of neighbouring genes originating from one or several genome regions. Each amplicon is supposed to bear at least one driver gene. Depending on the tumours, a few or most amplified genes may be over-expressed [5]–[10], so that several genes co-amplified within a given amplicon could be functionally relevant [11]–[13]. To determine the rationale for these differences in expression patterns, we analysed the expression level of all the co-amplified genes in five xenografted and one fresh human gliomas containing dmins, the sequence of which has been previously analysed in detail [14], [15]. We found that the tissue-specific pattern of gene expression is not modified upon amplification. In contrast, protein accumulation and/or activity are not tightly related to mRNA over-expression.

## Results

**Figure 1:**
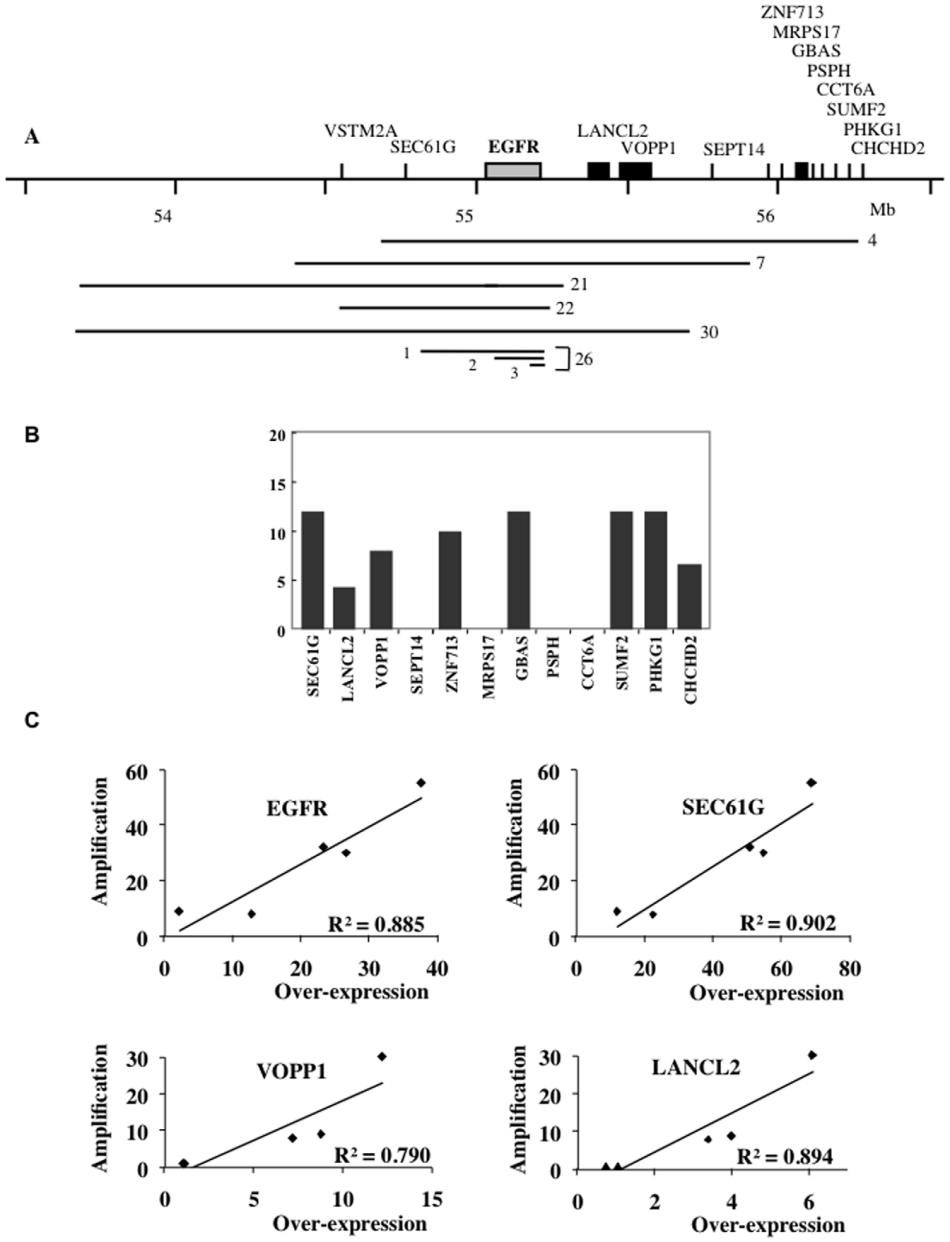
Relationships between gene copy number and mRNA expression in gliomas amplified for the 7p11–p12 locus. Full-length names of the genes are listed in Supporting Information Table S1. A. Position of the genes co-amplified with EGFR. The extent of the amplified regions is mapped for each glioma. In tumour 26, 3 amplicons of different sizes were present. B. Over-expression of genes co-amplified with EGFR in tumour 4 analysed by RT-Q-PCR. The expression ratio (R_e_) was calculated by dividing the normalised expression measured in the tumour by the mean of the normalised expressions measured in the reference set of tumours. Genes SEPT14, MRPS17, PSPH and CCT6A are expressed neither in the analysed glioma nor in the reference set. C: Relationships between amplification and mRNA expression for genes amplified in several tumours. EGFR and SEC61G were amplified in tumours 4, 7, 21, 22 and 30; LANCL2 and VOPP1 in tumours 4, 7 and 30. Primers used for EGFR detected the full-length and the deleted (EGFRvIII) forms of the gene.

In five out of the six gliomas we analysed (tumours 4, 7, 21, 22 and 30) the amplification process involved sequences originating only from the 7p11 region, wherein lies the EGFR gene. We first focussed on these simple situations, in which the amplicons range from 0.7 to 2.1 megabases depending on the tumour, and all include the EGFR gene [14] (Figure 1A). In addition, a rearrangement leading to deletion of EGFR exons 2 to 7, which is commonly observed in gliomas [16], was seen in all the amplicons of tumour 21 [14] and in about 20% of those of tumour 22 (Supplementary data S1). The deleted genes encode the well-known variant III of EGFR (EGFRvIII) [17], a constitutively active EGFR protein. Screening of the human reference genome sequence revealed that 14 genes coding for proteins lie in the region extending from 53 to 56.2 Mb, which overlaps all the sequences amplified in this set of tumours (Figure 1A, Supplementary Table S1). Among these genes, the SEC61G gene was co-amplified with EGFR in 5 tumours and the VSTM2A gene in 4 of them. Two genes (LANCL2 and VOPP1) and SEPT14 were respectively amplified in 3 and 2 tumours. Eight genes centromeric to SEPT14 (ZNF713, MRPS17, GBAS, PSPH, CCT6A, SUMF2, PHKG1 and CHCHD2) were amplified in tumour 4 only. The number of genes co-amplified with the EGFR gene thus ranges from 2 (tumour 22) to 12 (tumour 4).

The expression of these 14 genes was measured by RT-Q-PCR in tumours 4, 7, 21, 22 and 30 and in a reference set of 5 non-amplified gliomas (for an example of the procedure, see Supplementary data S2). The expression of 5 genes (VSTM2A, SEPT14, MRPS17, PSPH and CCT6A) was undetectable in all these gliomas, regardless of their amplification status. The other genes were expressed both in control and amplified tumours, and always over-expressed when amplified (Supplementary Table 1). In tumour 4, for example, 8 expressed genes were co-amplified with the EGFR gene. We found that these genes were over-expressed 4- to 12-fold, which agrees with their 9-fold amplification (Figure 1B). It is noticeable that original tumour 4 was available in addition to the xenograft, and that similar levels of expression were found in both cases (not shown). The fact that tumours with different copy numbers of a given gene are available offered us the possibility to determine the relationships linking the level of amplification to that of mRNA expression. We observed a linear relationship between these two parameters for the EGFR, SEC61G, VOPP1 and LANCL2 genes (Figure 1C), which shows that the level of mRNA expression correlates tightly with the gene copy number for expressed genes.

**Table 1:** Relationships between EGFR gene amplification and mRNA and protein accumulation.

Characteristic	Case 4	Case 7	Case 21	Case 22	Case 26	Case 30
Histology^a^	ODA	GBM	GBM	ODA	GBM	GBM
DNA amplification^b^	9	8	32	55	250^c^	30
mRNA over-expression d	8	13	24	38	300	27
Protein over-expression^e^	10	120	40	50+50^f^	150+100^f^	NA^g^
pEGFR form	wt	wt	vIII	wt+vIII	vI	NA^g^

**Figure 2:**
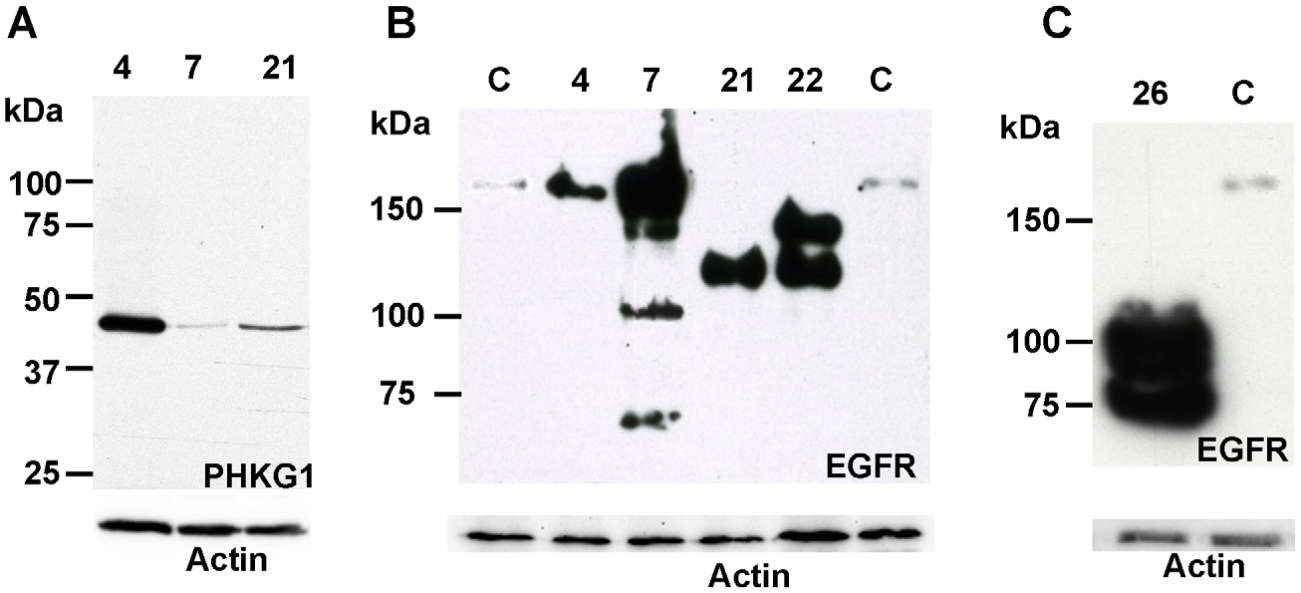
Western blot analysis of proteins encoded by amplified genes. A. Over-expression of PHKG1. The gene was amplified in tumour 4 and not amplified in tumours 7 and 21. B. Over-expression of EGFR. Controls (C): extracts from 2 glioblastomas without EGFR amplification expressing the wild-type form of the protein. Tumours 4 and 7: full-length wild-type EGFR. Tumour 21: EGFRvIII variant. Tumour 22: EGFRvIII variant plus full-length wild-type (the apparent small size of the wild-type form, also present as a minor product in tumour 7, could correspond to a protein with posttranslational modifications). Receptor degradation products are visible in the tumour 7 lane. C. Over-expression of EGFR in tumour 26. The two truncated proteins expressed in this tumour are of the EGFRvI variant form. Actin: loading control.

ODA: oligodendroglioma; GMB: glioblastoma multiforme.

a Histological type.

b mean level of amplification.

c amplicon 3, unexpressed amplicons 1 and 2 were both amplified 8-fold

d Mean level of mRNA expression.

e Mean value of protein accumulation.

f The 2 bands were quantified independently (see Figure 2).

g protein extract not available.

Protein extracts were available for tumours 4, 7, 21 and 22 but exhaustive analysis of the accumulation of proteins encoded by amplified genes was prevented by the lack of adequate antibodies. However, it was possible to perform western-blot analysis of PHKG1 and EGFR. In the case of PHKG1, we found that the protein was more abundant in cells of tumour 4 than in cells of tumours without amplification of that gene (Figure 2A). For EGFR, we observed a band around 170 kDa in extracts from tumours 4 and 7 that corresponds to the normal position of EGFR (Figure 2B). Cells of tumour 21 accumulated a protein of about 130 kDa which corresponds to EGFRvIII. Two proteins migrating at 145 and 130 kDa were detected in tumour 22. The latter band corresponds to EGFRvIII which was encoded by about 20% of the amplicons (see above). Even though the 145 kDa protein migrated faster than the normal EGFR, sequencing of the whole EGFR cDNAs indicated that no deletion affected the EGFR full-length allele present in the rest of the amplicons (not shown). This result suggests that post-translational modifications might be responsible for the electrophoretic shift also observed for a fraction of the protein in tumour 7 (Figure 2B). Quantification of the intensity of the bands (Table 1) showed that the EGFR protein was always more abundant in tumours where the gene was amplified than in control tumours, but the relationship between EGFR mRNA and protein accumulation was loose in some tumours. For example, tumours 4 and 7 had a very similar gene copy number and mRNA over-expression but showed an around ten-fold difference in protein accumulation. In addition, the relative amount of wild type and variant proteins do not parallel the copy number of the corresponding genes.

**Figure 3:**
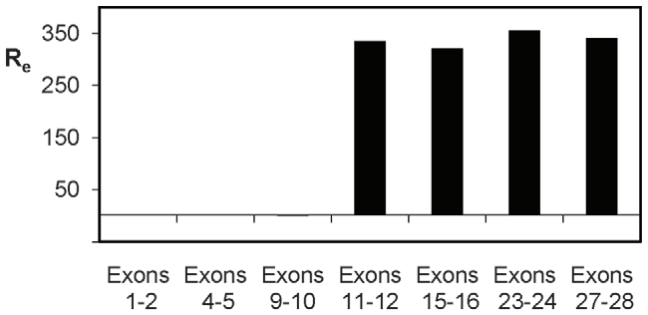
RT-Q-PCR analysis of the expression along the EGFR gene in tumour 26. For each pair, the forward and reverse primers were localised in consecutive exons in order to prevent amplification of DNA traces present in the mRNA preparations. The expression ratio (R_e_) was calculated by dividing the normalised expression measured in the tumour by the mean of the normalised expressions measured in the reference set of tumours. Exons 1 to 10 were not over-expressed, indicating that only the truncated form of the gene coded by amplicon 3 was expressed, whereas the large forms present in amplicons 1 and 2 were not expressed (see Figure 1).

Analysis of tumour 26 have revealed a complex situation, since five types of amplicons were identified [15] (Figure 1A, Supplementary Figure S1). Amplicons 1 to 3 contained fragments from the EGFR locus. The gene was full-length in amplicon 1, the region containing the first exon of the gene was missing in amplicon 2, and only exons 11 to 28 were present in amplicon 3 (Details on the amplicon structures are available in supplementary Figure S1). Only exons 11 to 28 were detected by RT-Q-PCR analysis, indicating that only the truncated gene of amplicon 3 was expressed in this tumour (Figure 3, Supplementary data S3). In this amplicon, the fragment from the EGFR locus was associated with a fragment from chromosome 5 (Supplementary Figure S1). PCR and RT-Q-PCR scanning of chromosome 5 and 7 fragments present in amplicon 3 showed that a sequence longer than 150 kb, containing the processed mRNA of the truncated EGFR gene, was transcribed from this amplicon (Supplementary data. S4). This transcript seems unusually long, but a global analysis (ENCODE project) has previously suggested that such long-distance transcription products are present in primary nuclear transcripts [18]. Western blotting revealed the presence of two EGFR variants of 75 and 90 kDa (Figure 2C) likely corresponding to the two forms observed at the mRNA level. These truncated variants lacking the extracellular part of the wild-type protein (Supplementary Data S5) are similar to the constitutively active variant I of EGFR (EGFRvI) [19]. Reanalysis of data previously published by Wong et al. [20], allows us to suggest that the formation of an extra-chromosomal circular DNA molecule could be a recurrent mechanism in the amplification and over-expression of the EGFRvI variant in gliomas (Supplementary Data S6).

**Figure 4:**
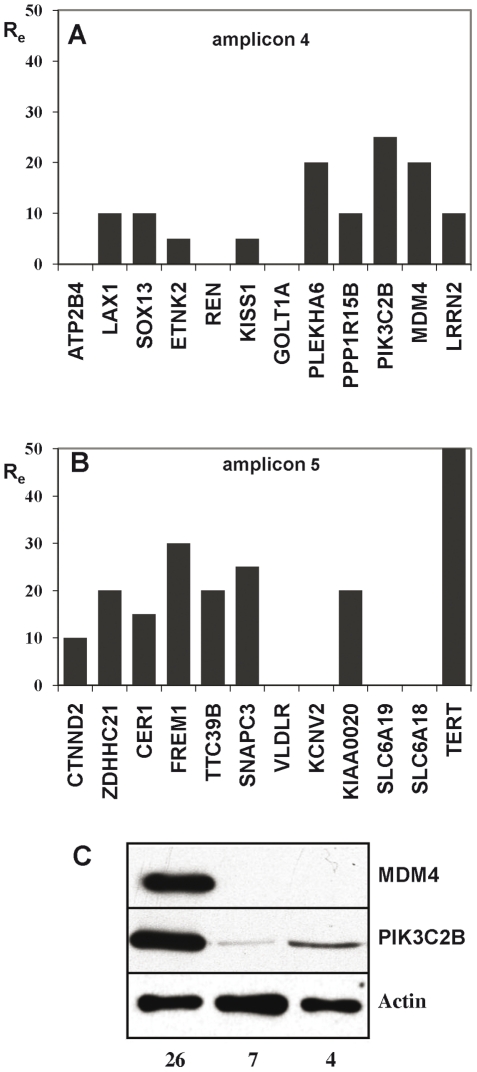
RT-Q-PCR expression of genes amplified in tumour 26. Full-length names of the genes are listed in Supporting Information Table S1. A: Amplicon 4; B: Amplicon 5. Positions of the genes are shown in Supporting Information Fig. S1. The expression ratio (R_e_) was calculated by dividing the normalised expression measured in the tumour by the mean of the normalised expressions measured in the reference set of tumours. ATPB4, REN, GOLT1A, VLDLR, KCNV2, SLC6A19 and SLC6A18 are expressed neither in tumour 26 nor in the reference set. C. Western blot probed with the anti-MDM4 and anti-PIK3C2B antibodies for gliomas 26, 7 and 4. Proteins were over-expressed in tumour 26. Actin: loading control.

In addition to the 3 amplicons containing the EGFR locus, two other amplicons were present in tumour 26. We have previously shown that amplicon 4 corresponds to the circularisation of a single fragment from 1q32.1 while amplicon 5 results from the complex association of 4 fragments from different regions of chromosome 5 and 2 fragments from chromosome 9 [15] (Details on the amplicon structures are available in supplementary Figure S1). We analysed the expression of 24 genes present on these amplicons, the others being pseudogenes or truncated genes with a few amplified exons (Supplementary Table S1). Seventeen genes were found to be over-expressed, 5- to 25-fold for those amplified 10 times (amplicon 4) and 10- to 50-fold for those amplified 15 times (amplicon 5) (Figure 4 A and B). The level of expression of the 7 remaining genes was under the threshold of detection in tumour 26 and in the 5 tumours of the reference set (Figure 4 A and B). Antibodies were available for two of the over-expressed genes, MDM4 and PIK3C2B. Western blot analyses showed that both proteins accumulate in tumour 26 as compared with tumours without amplification of the genes (Figure 4C).

The telomerase TERT gene was found over-expressed about 50-fold in tumour 26 but the level of protein accumulation could not be evaluated as none of the currently available antibodies allow efficient detection of the protein on western blot ([21] and unpublished data). No increase in TERT activity was observed in this tumour as compared with tumours without amplification of the gene (Supplementary data S7). No deletion, insertion or alternative splicing that could generate an inactive protein was detected by sequencing the whole coding sequence of the mRNA encoding the normal isoform 1 of the protein (not shown). In addition, the RNA component of the telomerase (TERC) was expressed at very low level in this tumour as compared with the tumours of the control set (Supplementary data S7). Moreover, we found that the telomeres were shorter in tumour 26 (less than 5 kb) than in two gliomas without TERT amplification, which display normal telomere lengths of 5 to 15 kb (Supplementary data S7).

## Discussion

Gene amplification plays a pivotal role during malignant transformation when conferring a growth advantage to the cells. Most of the time the amplicons are very large and contain many genes, more than one of which may have a biological significance for tumour development. For example, EGFR is generally considered as the driver gene at 7p11 [22], however, SEC61G is recurrently co-amplified and over-expressed in glioma ([23], [24] and Figure 1A). SEC61G encodes a subunit of a heterotrimeric protein channel located in the endoplasmic reticulum and it has been shown that silencing of SEC61G expression in glioblastoma cells led to growth suppressiio and apoptosis [25]. Thus, in addition to EGFR, SEC61G is an amplification target in a large fraction of the gliomas. In order to get more insight into the final outcome of amplification, we investigated the relationships between gene amplification, mRNA over-expression and protein accumulation in a series of gliomas with well-characterised extra-chromosomal amplicons [14], [15]. Even though our work is limited to six cases and a single tumour type, several of our findings could be of general interest.

Not all amplified genes are over-expressed in the gliomas we studied. We found that 5 out of 14 genes from the EGFR locus are not expressed when amplified. Strikingly all five genes are also not expressed in gliomas with a normal copy number of the locus (Figure 1B and supplementary Table S1). In addition to sequences from the EGFR locus, tumour 26 displays complex amplicons containing fragments from chromosomes 1, 5, 7 and 9. Seven out of 17 amplified genes originating from the latter chromosomes are expressed neither in this tumour nor in the set of non-amplified gliomas. Applying the hypergeometric law, the probability that the 12 unexpressed genes in a series of 38 genes remain unexpressed after amplification only by chance is of 3.7×10^−10^. This strongly suggests that maintenance of tissue-specific gene extinction is a general occurrence in these tumours (Figures 4 A and B). In the case of expressed genes, their mRNA appeared to be always more abundant in amplified cells than in cells non-amplified for the corresponding gene. However, we found that different genes co-amplified on the same amplicon are not necessarily over-expressed at identical levels (Figures 1B, 4A and B). This is likely due to individual variations in gene expression, a phenomenon also observed in the reference set of non-amplified tumours (Supplementary data S2). Nevertheless, the results obtained with tumours arboring different copy numbers of the EGFR, SEC61G, LANCL2 and VOOP1 genes allow us to establish that mRNA over-expression parallels the level of amplification (Figure 1C). Thus, normal gene expression profiles are generally retained upon amplification. Determination of the level of protein accumulation, when possible, also showed that proteins are more abundant in tumours in which the mRNA is over-expressed than in tumours without over-expression (Table 1 and Figures 2 and 4), but the correlation is far less close than between copy number and mRNA over-expression. For example, gliomas with similar EGFR gene copy number and mRNA over-expression may accumulate quite different levels of the corresponding protein (Table 1).

It has long been known that the level of TERT mRNA is elevated in high-grade gliomas as compared with low-grade gliomas, and that high expression of the gene is associated with a significant increase in telomerase activity [26], [27]). Whether over-expression of the TERT gene results from gene amplification was not investigated in these tumours. A more recent study showed that amplification and over-expression of both TERT and TERC occur in leukemic cells [28]. In tumour 26, the mRNA of the normal TERT isoform 1 was over-expressed at a high level but TERC was weakly expressed. The lack of antibodies able to specifically recognise the reverse transcriptase prevented us from evaluating the level of protein present in these cells but *in vitro* assays showed no increase in TERT activity. In addition, we found that telomeres are rather short in cells of tumour 26 as compared with tumours without TERT amplification, suggesting that TERT protein does not accumulate in tumour 26 or that the RNA component of telomerase is limiting [29]. Thus, if TERT gene over-expression plays a role in the development of that tumour, it should be in a telomere length-independent manner [30].

The actual impact of amplification of a given gene on glioma cell biology is therefore difficult to predict from transcriptome analysis only. Our results suggest that translational and/or post-translational regulation plays a major role in the control of protein accumulation. Besides, protein accumulation could be unlinked from protein activity in many cases. Altogether, these features may minimise the contribution of gene copy number to the physiological impact of gene amplification.

## Materials and Methods

### Biological material

The 6 tumours (4 glioblastomas multiforme and two oligodendrogliomas) were described previously [14], [15], [31] (Table 1). Xenografted tumours were used for cases 4, 7, 21, 22 and 26. The corresponding fresh tumour was also studied for case 4. The fresh tumour only was available for case 30. Five xenographted glioblastomas without amplification were used as reference set.

### DNA and RNA analysis

DNA and RNA preparation and characterisation, sequencing procedures and PCR methods were described previously [14], [15].

### Expression analysis

Gene locations and sequences used in this work refer to the human reference genome sequence (released March 2006) which is available at the University of California, Santa Cruz Genome Bioinformatics website (http//genome.ucsc.educ) [32]. The mRNA level was measured by real-time fluorescent quantitative RT-PCR. cDNAs were prepared using the SuperScript II system according to the manufacturer's protocol (Invitrogen) and amplified using the GeneAmp 7500 sequence detection system and SYBR Green PCR Kits (Applied Biosystems). The specificity and the efficiency of the primers were tested using a mixture of RNA from several cells lines. Primer sequences are available on request. Gene expression was normalised using the 3 housekeeping genes, the TATA box binding protein (TBP), beta-actin (ACTB) and DNA-directed RNA polymerase II polypeptide A (POLR2A), determined as described [33]. For each gene, the expression ratio (R_e_) was calculated by dividing the normalised expression measured in the tumour by the mean of the normalised expressions measured in the reference set of tumours. Each point was the mean value of three independent experiments. A gene was considered as unexpressed when the RT-Q-PCR curve was indistinguishable from the background (Ct>38).

### Western blotting

Protein extraction and Western blot analysis were performed as described previously [34]. Briefly, tumour tissues were solubilised in Laemmli buffer and protein samples (20 µg) were subjected to SDS/PAGE gel electrophoresis followed by electro-transfer on an Immobilon-P membrane (Millipore). Rabbit polyclonal anti-EGFR (sc-03, Santa Cruz), rabbit polyclonal anti-PHKG1 (RB3704, Abgent), rabbit polyclonal anti-MDM4 (A300-287A, Bethyl), mouse monoclonal anti-PIK3C2B (H00005287-M0, Abnova 2 and mouse monoclonal anti-actin (sc-47778, Santa Cruz) were used. HRP-conjugated goat anti-mouse-IgG or anti-rabbit-IgG secondary antibodies (Dako) were used along with the Pierce Supersignal West Pico Chemiluminescence substrate. Protein abundance was estimated by densitometry of the films at several exposure times. The level of accumulation was determined by comparison with the mean value of the expression of gliomas without amplification of the gene.

### Telomerase activity

Telomerase (TERT) activity was measured after extraction of the proteins in CHAPS buffer (TRAPeze CHAPS Lysis buffer, Chemicon) as described [35]. The RNA component of telomerase (TERC) was quantified by RT-Q-PCR [36]. Telomere length was determined by Southern blot [37].

## Supporting Information

Data S1 Detection of EGFRvIII in tumour 22.
(1.18 MB DOC)

Data S2 Quantitative RT-PCR analysis of the genes of the EGFR locus.
(0.49 MB DOC)

Data S3 Expression of EGFR in tumour 26.
(0.03 MB DOC)

Data S4 Processing of the EGFR gene in amplicon 3 of tumour 26.
(0.37 MB DOC)

Data S5 Structure of the EGFRvI proteins encoded by amplicon 3.
(0.03 MB DOC)

Data S6 Structure of the amplified EGFR mRNA published by Wong et al.
(0.05 MB DOC)

Data S7 Telomerase expression.
(0.35 MB DOC)

Figure S1 Structure of the 5 amplicons present in tumour 26.
(0.49 MB DOC)

Table S1 List of the genes amplified in the analysed tumour. Red, overexpressed genes. Blue, genes not over-expressed. Black, not analysed: pseudogenes or truncated genes with a few amplified exons.
(0.04 MB DOC)
