# Supplementary material for: Relationships Linking Amplification Level to Gene Over-Expression in Gliomas
Source: PLoS One. 2010 Dec 8;5(12):e14249. doi: 10.1371/journal.pone.0014249 (PMC2999539; doi:10.1371/journal.pone.0014249)
Supplement: Data S1 — Detection of EGFRvIII in tumour 22. (1.18 MB DOC) [file pone.0014249.s001.doc]

**Supplementary Information data S1**

**Detection of EGFRvIII in tumour 22.**

The deletion of exons 2-7 of the EGFR gene, which is the cause of the EGFRvIII variant formation, was not detected in our previous analysis of tumour 22 genomic DNA using quantitative PCR, whereas the deleted variant amplified in tumour 21 was found [1]. However, the presence in tumour 22 of the variant form of the protein beside the wild-type form (see Figure 3 in results) prompted us to re-analyse the EGFR gene in this tumour. We first analysed the structure of the mRNA using RT-PCR. Primers located in exons 1 and 8 (Forward: 5' TGACTCCGTCCAGTATTGATCG 3'; Reverse: 5' GCCCTTCGCACTTCTTACACTT 3') were used to amplify the cDNA region containing exons 2 to 7 or the deletion (Figure 1). The full-length cDNA fragment (1049 bp) was obtained with the breast cancer cell line SK-BR3 and tumours 7 and 4, which expressed the wild-type protein. In contrast, tumours 21 and 22 displayed both the PCR fragment corresponding to the wild-type (1049 bp) and the deleted (248 bp) forms. The lack of exons 2-7 was confirmed by sequencing of the short fragment (not shown). These data suggested that beside the wild-type form, a fraction of the amplified EGFR gene had a deletion of exons 2-7.

To confirm this hypothesis, we used a PCR-based strategy developed for the characterisation of rearranged fragments associated with the EGFRvIII variant receptor [2]. DNA from tumours 21 and 22 was subjected to long-range PCR using a single antisense primer located in the eighth exon of the gene, and one sense primer located in intron 1. A series of 27 sense primers were tested to locate the position of the deletion. Because of the distance between the upstream and downstream primers, obtaining a PCR product was possible only in the presence of a large deletion. Sequencing of the amplified fragment allows the location of the deletion to be determined. The sequences of the primers used are listed in [2]. Using this approach, the deletion previously observed in tumour 21 was found again (not shown) and a deletion was characterised in tumour 22. The deletion of 93,858 bp was located between positions 55,096,402 (intron 1) and 55,190,260 (intron 7) on chromosome 7. We determined the percentage of amplified fragments harbouring the deletion using quantitative PCR. Primer pairs were selected in the amplified EGFR gene, both in the deleted sequence and outside of this region. These primer pairs were used to measure the full-length and full-length plus the deleted sequences present in the DNA of the tumour, respectively. About 20% of the deleted form was present (not shown).

The fusion of the two breakpoints introduced in the EGFR gene by the deletion produced a complex junction. It is characterised by the insertion of a fragment of 89 bp from the distal region of the EGFR gene (positions 54,717,259 - 54,717,348), flanked in 5' and in 3' of a sequence of 13 and 18 bp, respectively (Figure 2). No homology was found in the human genome for the 18 bp sequence. The 13 bp sequence was present in 5p14.3, 10p11.23, 6q25.3 and 3q25.33, however the significance of homologies between such short sequences is questionable. These sequences could be formed during fragment fusion by nontemplated DNA synthesis. No significant homologies were found at the breakpoints The complex structure of the junction of the deletion in the EGFRvIII variant found in tumour 22 suggests the that a secondary rearrangement of an already extrachromosomal DNA molecule is involved in the formation of this deleted form.

References.

1. Vogt N, Lefevre SH, Apiou F, Dutrillaux AM, Cor A, et al. (2004) Molecular structure of double-minute chromosomes bearing amplified copies of the epidermal growth factor receptor gene in gliomas. Proc Natl Acad Sci U S A 101: 11368-11373.

2. Frederick L, Eley G, Wang XY, James CD (2000) Analysis of genomic rearrangements associated with EGRFvIII expression suggests involvement of Alu repeat elements. Neuro-oncol 2: 159-163.


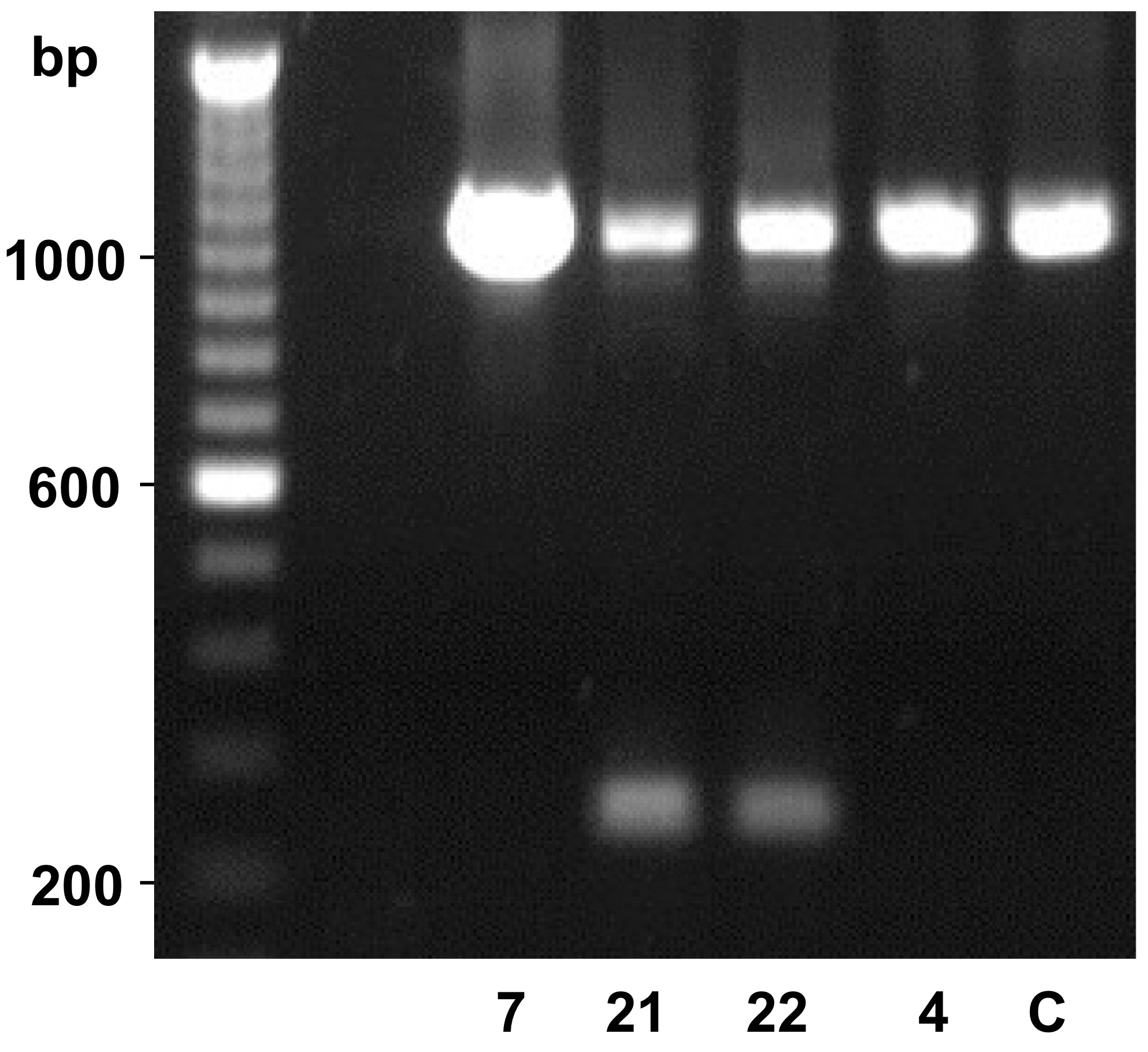


**Figure 1: Presence of the EGFRvIII form in tumours 21 and 22.** Agarose gel analysis of the RT-PCR products: full-length cDNA, 1049 bp; deleted cDNA, 248 bp. C: control SKBR3 cell line.

GCTCTTCATG ACTGTATCTG TACCAGCAAT ATTTGTATTG GCAAATCACA 55096303

TGCCCCAGTG GGAACTACTT AAGGGGAATT CAATGGATTT CATTCCTTTT 55096353

ATGTAATTGG CCACTTAGTA ATAGACGTGT AGGTCTCTTG TGTGGATAAa 55096403

aataatggtt tcatatttag gaaaagctaa atatgttcaa aaaatggata

atatctcaat tgctatgtag gaaaataact atagcatatc aaaatttaca

tattttagag ctccatttaC AGATGGTTCA CAAAGAATCA TCAATGTCCT 55190293

ATAAATATTT TTGAGGATCT TCTTGGGGAA CTTAAAACAG GAACAGCCAG 55190343

**Figure 2. Sequence of the junction of the deletion in the EGFRvIII form.** Blue: intron 1 (upper part) and intron 7 (lower part) of the EGFR gene; Red: insertion of a fragment from a region distal of the gene; Black: flanking sequences.
