# Supplementary material for: Relationships Linking Amplification Level to Gene Over-Expression in Gliomas
Source: PLoS One. 2010 Dec 8;5(12):e14249. doi: 10.1371/journal.pone.0014249 (PMC2999539; doi:10.1371/journal.pone.0014249)
Supplement: Data S2 — Quantitative RT-PCR analysis of the genes of the EGFR locus. (0.49 MB DOC) [file pone.0014249.s002.doc]

**Supplementary Information S2**

**Quantitative RT-PCR analysis of the genes of the EGFR locus.**

RT-Q-PCR of the genes were performed in the same experiment for the cases 4, 7, 21, 22, 30 and the reference set of gliomas without amplification (A - E). Each RT-Q-PCR was done in duplicate, and the mean value of the Ct was calculated.

Five genes of the locus, VSTM2A, SEPT14, MRPS17, PSPH and CCT6A were not expressed (RT-Q-PCR curve indistinguishable from the background, Ct> 38).

For the 9 expressed genes, the tumour with the highest expression (the lowest Ct value) was selected as reference tumour. The deltaCt was calculated by subtracting the Ct of the reference tumour from the Ct of each tumour.

delta Ct = Ct(tumour) - Ct(reference)

Relative quantities were obtained from the deltaCt using the formula

Quantity = 1/2 deltaCt

The normalised relative quantities were obtained by dividing the quantities by the normalisation factor obtained using 3 housekeeping genes (see Material and Methods).

Normalised relative quantities are presented in Tables 1 (reference set) and 2 (analysed gliomas). The figure presents the synthesis of the data.

For each gene, the over-expression was calculated by dividing the normalised relative quantity measured for each tumour by the mean value of the normalised relative quantities obtained for the tumours of the reference set.

The same experiment was performed 3 times. Data presented in Figure 1 of the text are the mean values of these 3 independent experiments.

Table 1

| case | SEC61G | EGFR | LANCL2 | VOPP1 | ZNF713 | GBAS | SUMF2 | PHKG1 | CHCHD2 |
| --- | --- | --- | --- | --- | --- | --- | --- | --- | --- |
| A | 0.027 | 0.017 | 0.082 | 0.01 | 0.027 | 0.048 | 0.027 | 0.09 | 0.107 |
| B | 0.013 | 0.008 | 0.075 | 0.057 | 0.005 | 0.032 | 0.019 | 0.057 | 0.05 |
| C | 0.018 | 0.012 | 0.06 | 0.032 | 0.013 | 0.084 | 0.01 | 0.013 | 0.032 |
| D | 0.015 | 0.01 | 0.015 | 0.026 | 0.007 | 0.024 | 0.013 | 0.04 | 0.078 |
| E | 0.007 | 0.003 | 0.048 | 0.02 | 0.008 | 0.012 | 0.021 | 0.01 | 0.018 |
| **Mean** | **0.016** | **0.01** | **0.056** | **0.029** | **0.012** | **0.04** | **0.018** | **0.042** | **0.057** |

Expression of the genes of the EGFR locus in the gliomas of the reference set. Normalised relative quantities are presented for the 9 genes. Mean: for each gene, mean value of the normalised relative quantities .

Table 2

| case | SEC61G | EGFR | LANCL2 | VOPP1 | ZNF713 | GBAS | SUMF2 | PHKG1 | CHCHD2 |
| --- | --- | --- | --- | --- | --- | --- | --- | --- | --- |
| 4 | **0.211 (13.2)** | **0.022 (2.2)** | **0.23 (4.2)** | **0.281 (9.7)** | **0.102 ( 8.5)** | **0.528 (13.2)** | **0.221 (12.3)** | **0.936 (13)** | **0.478 5.5)** |
| 7 | **0.321 (20.1)** | **0.14 ( 14)** | **0.162 (2.9)** | **0.203 (7)** | 0.017 (1.42) | 0.061 (1.52) | 0.017 (0.95) | 0.045 (1.07) | 0.034 (0.6) |
| 21 | **0.757( 47.3)** | **0.287 (28.7)** | 0.059 (1.05) | 0.031 (1.07) | 0.013 (1.08) | 0.048 (1.21) | 0.011 (0.63) | 0.068 (1.62) | 0.072 (1.27) |
| 22 | **1.161 (72.6)** | **0.453 (45.3)** | 0.042 (0.75) | 0.033 (1.13) | 0.007 (0.57) | 0.03 (0.75) | 0.022 (1.22) | 0.048 (1.15) | 0.07 (1.23) |
| 30 | **0.91 (57)** | **0.201( 20.1)** | **0.453 (8.1)** | **0.409 (14.1)** | 0.008 (0.71) | 0.045 (1.12) | 0.034 (1.9) | 0.034( 0.82) | 0.054 ( 0.95) |

Expression of the genes of the EGFR locus in the 5 gliomas with amplifications. Normalised relative quantities are presented for the 9 genes. Bold: gene amplified in the tumour. The over-expression level is in brackets.


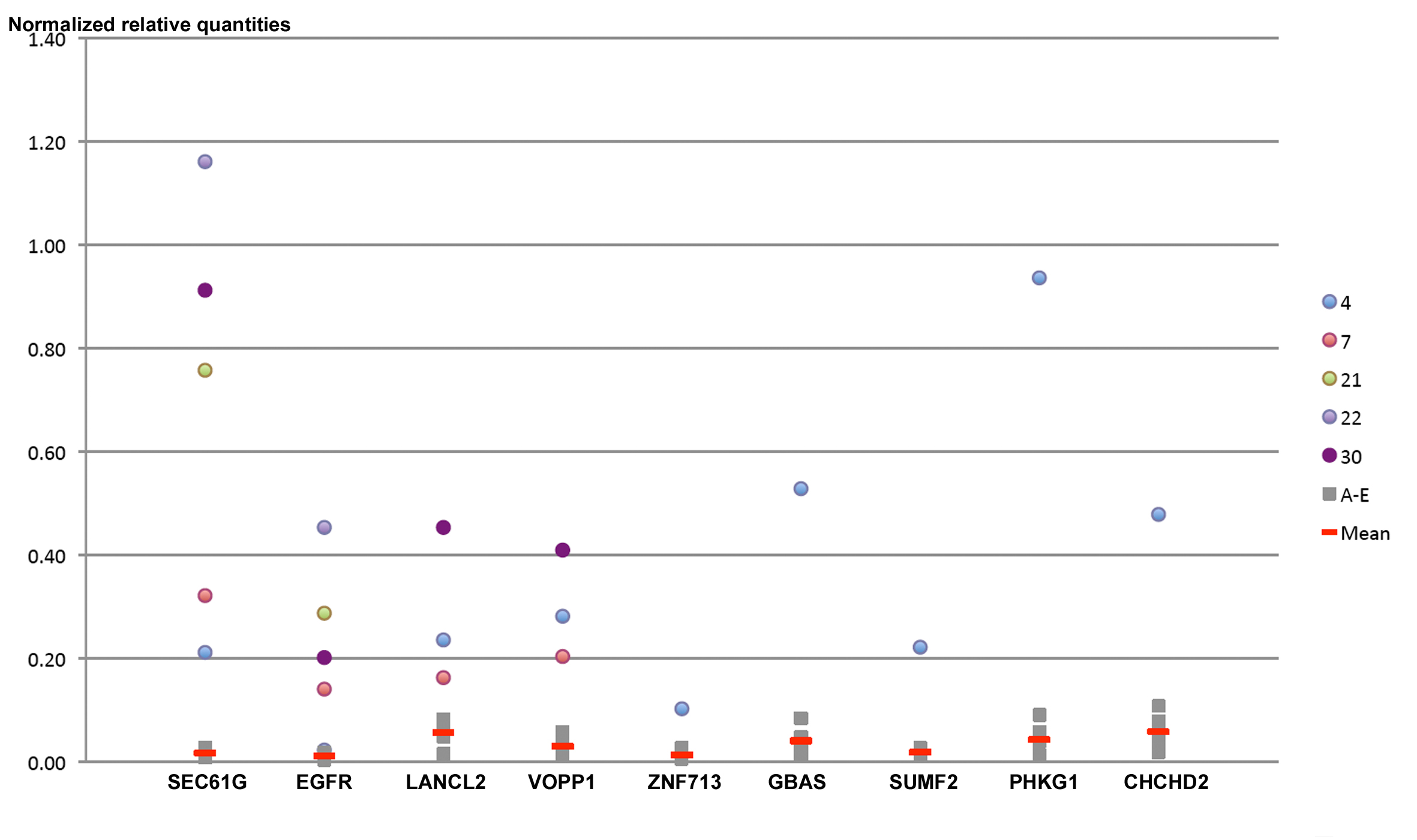


Expression of the genes of the EGFR locus in the analysed gliomas. Normalised relative quantities can be compared for a gene in different tumours but not between genes. Mean: for each gene of the reference set (tumour A -E), mean value of the normalised relative quantities used for the determination of the over-expression of the amplified genes in the gliomas 4, 7, 21, 22 and 30.
