# Supplementary material for: Relationships Linking Amplification Level to Gene Over-Expression in Gliomas
Source: PLoS One. 2010 Dec 8;5(12):e14249. doi: 10.1371/journal.pone.0014249 (PMC2999539; doi:10.1371/journal.pone.0014249)
Supplement: Data S3 — Expression of EGFR in tumour 26. (0.03 MB DOC) [file pone.0014249.s003.doc]

**Supplementary Information data S3**

**Expression of EGFR in tumour 26.**

The mRNA expression of the EGFR gene was analysed by RT-Q-PCR using primers located along the cDNA. No over-expression was detected using primer pairs located in exons 1 to 10, whereas exons 11 to 28 were over-expressed 300- to 350-fold (Fig. 3 in result). The lack of over-expression of exons 1 to 10 indicated that the two larger forms of the amplified EGFR gene were not expressed. The absence of the promoter region may explain the inactivation of the gene in amplicon 2, however in amplicon 1 the whole gene was amplified. No mutation was found by sequencing exons, intron-exon junctions or the promoter region. In addition deletion were not found by southern blotting (not shown). It has been shown that, in tumour cell lines, the transcription silencing of EGFR is closely correlated with the hypermethylation of the CpG island located at the 5' end of the gene that extends into exon 1 [1]. We used the same approach to compare the methylation status of this region in tumour 26 with control tumours: tumours 4 and 21, in which EGFR was amplified and over-expressed, and in two tumours of the reference set devoid of EGFR amplification. Sequencing of the bisulphite-treated DNA showed that, in the five tumours, none of the 15 CpG located in the promoter and exon 1 regions was methylated at a detectable level (not shown). So, in tumour 26, the lack of expression of the full length EGFR gene was not due to the hypermethylation of the promoter region

**Material and methods.**

The methylation of the CpG island of the EGFR gene was analysed as described [1] using the EZ DNA Methylation Kit (Zymo Research) for bisulphite treatments.

**References**

1. Montero AJ, Diaz-Montero CM, Mao L, Youssef EM, Estecio M, et al. (2006) Epigenetic inactivation of EGFR by CpG island hypermethylation in cancer. Cancer Biol Ther 5: 1494-1501.
