# Supplementary material for: Relationships Linking Amplification Level to Gene Over-Expression in Gliomas
Source: PLoS One. 2010 Dec 8;5(12):e14249. doi: 10.1371/journal.pone.0014249 (PMC2999539; doi:10.1371/journal.pone.0014249)
Supplement: Data S4 — Processing of the EGFR gene in amplicon 3 of tumour 26. (0.37 MB DOC) [file pone.0014249.s004.doc]

**Supplementary Information data S4**

**I. Processing of the EGFR gene in amplicon 3 of tumour 26.**

The junctions between the EGFR mRNA and the chromosome 5 sequences were analysed by RT-PCR using a primer located in exon 20 of the EGFR gene and the other in chromosome 5 (Figure 1). The length and the nucleotide sequence of the amplified fragment indicated normal processing of the exons of the 3' mRNA region of the EGFR gene and the continuation of the transcript up to the chromosome 5 sequence (Figure 1 and not shown). The 5' mRNA region was analysed using a single primer located upstream of the junction between chromosomes 5 and 7 and primers located in exons 12, 15 and 20 of EGFR, respectively (Figure 1). A large amplified fragment was obtained in all cases, whereas with primers located in exons 15 and 20, a smaller fragment was also observed. Sequencing the large amplified fragments established that the processed exons of EGFR were associated with a truncated part of the intron 10 and with the chromosome 5 sequence (not shown). The small fragments correspond to the same sequence devoid of exons 12 to 14. No deletion in the genomic DNA was observed in this region by long-range PCR, indicating that an alternative splicing caused the deletion of the exons (not shown).


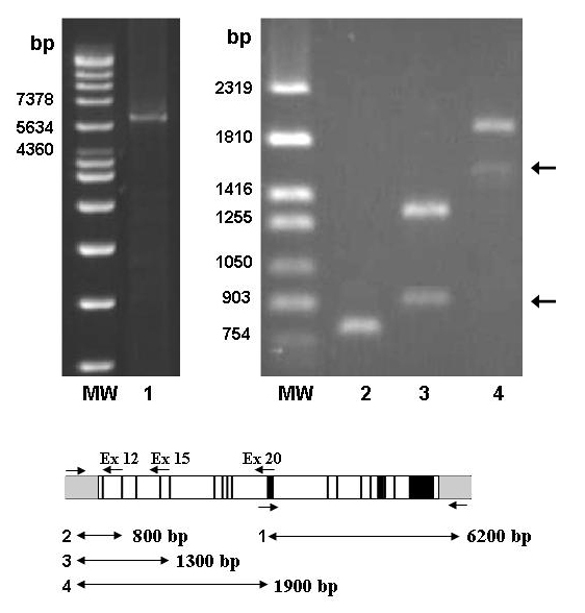


**Figure 1**

RT-PCR analysis of the transcripts. 1: between the chromosome 5 segment in 3' of the EGFR gene and exon 20. 2, 3 and 4: between the chromosome 5 segment in 5' of the EGFR gene and exons 12, 15 and 20, respectively. Fragments were analysed using agarose gel electrophoresis. The lengths of the processed transcripts are indicated. The expected transcript was observed with all primer pairs; in 3 and 4, another smaller band (arrows) was observed corresponding to a transcript devoid of exons 12-14.

**II. Transcription of the amplicon 3.**

The whole chromosome 5 segment of the amplicon 3 was screened using RT-Q-PCR to determine the extent of the expressed sequences both sides of the EGFR gene (Figure 2A). In 3' of the EGFR segment, chromosome 5 sequences were transcribed on about 11 kb, whereas in 5', mRNA formation was observed on more than 145 kb. These sequences were expressed at the same level as the EGFR mRNA. The Q-PCR performed in parallel using the RNA preparation without reverse transcription failed to show amplification above the background, indicating that a putative DNA contamination of the sample did not introduce a bias in the analysis of the expression. Both 3' and 5' rapid amplifications of cDNA ends (SMART RACE cDNA amplification kit, Clontech) were performed to determine the polyadenylation and cap sites, respectively. The 3' polyadenylation site was in the CA dinucleotide at position 11,843,828 on chromosome 5. A signal sequence, AATAAA, was present 14 bp upstream of the polyadenylation site. Experiments failed to find the cap in 5', but it was established that the over-expressed sequence ended between positions 11,642,000 and 11,642,300. A northern blot probed with a RT-PCR fragment of the over-expressed sequence displayed a band in the non-resolving region of the gel, associated with a smear of degradation products (Figure 2B).


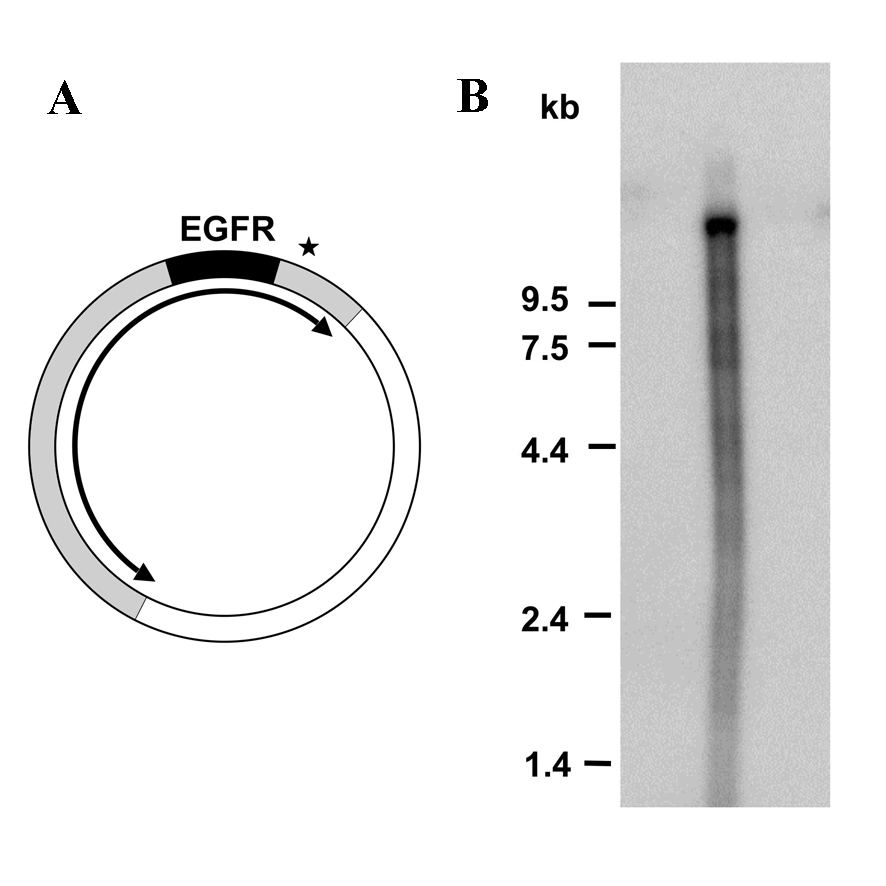


Figure 2.

Transcription of the amplicon 3 from tumour 26. A. Arrow: extent, determined by quantitative RT-PCR, of the extrachromosomal DNA expressed region; star: position of the probe used for the northern blot. B. Northern blot of total RNA using the probe from the expressed region of amplicon 3, a high-molecular-weight band located in the non-resolving region of the gel was associated with a smear of degradation products.
