# Supplementary material for: Relationships Linking Amplification Level to Gene Over-Expression in Gliomas
Source: PLoS One. 2010 Dec 8;5(12):e14249. doi: 10.1371/journal.pone.0014249 (PMC2999539; doi:10.1371/journal.pone.0014249)
Supplement: Data S5 — Structure of the EGFRvI proteins encoded by amplicon 3. (0.03 MB DOC) [file pone.0014249.s005.doc]

**Supplementary Information data S5**

**Structure of the EGFRvI proteins encoded by amplicon 3.**

G I A A E G G R Q L Stop T V A R P A S A L C T L R E D D N E K E S K S N F P T Y C Y N T E S L R V Stop S N V H I I Q S P Stop E S R V **Met** S H T K K K L L R G V C L K S F I C L T G F L L I Q A W P E N R T D L H A F E N L E I I R G R T K Q H G Q F S L A V V S L N I T S L G L R S L K E I S D G D V I I S G N K N L C Y A N T I N W K K L F G T S G Q K T K I I S N R G E N S C K A T G Q V C H A L C S P E G C W G P E P R D C V S C R N V S R G R E C V D K C N L L E G E P R E F V E N S E C I Q C H P E C L P Q A Met N I T C T G R G P D N C I Q C A H Y I D G P H C V K T C P A G V Met G E N N T L V W K Y A D A G H V C H L C H P N C T Y G C T G P G L E G C P T N G P K I P S I A T G Met V G A L L L L L V V A L G I G L F Met R R R H I V R K R T L R R L L Q E R E L V E P L T P S G E A P N Q A L L R I L K E T E F K K I K V L G S G A F G T V Y K G L W I P E G E K V K I P V A I K E L R E A T S P K A N K E I L D E A Y V Met A S V D N P H V C R L L G I C L T S T V Q L I T Q L Met P F G C L L D Y V R E H K D N I G S Q Y L L N W C V Q I A K G Met N Y L E D R R L V H R D L A A R N V L V K T P Q H V K I T D F G L A K L L G A E E K E Y H A E G G K V P I K W Met A L E S I L H R I Y T H Q S D V W S Y G V T V W E L Met T F G S K P Y D G I P A S E I S S I L E K G E R L P Q P P I C T I D V Y Met I Met V K C W Met I D A D S R P K F R E L I I E F S K Met A R D P Q R Y L V I Q G D E R Met H L P S P T D S N F Y R A L Met D E E D Met D D V V D A D E Y L I P Q Q G F F S S P S T S R T P L L S S L S A T S N N S T V A C I D R N G L Q S C P I K E D S F L Q R Y S S D P T G A L T E D S I D D T F L P V P E Y I N Q S V P K R P A G S V Q N P V Y H N Q P L N P A P S R D P H Y Q D P H S T A V G N P E Y L N T V Q P T C V N S T F D S P A H W A Q K G S H Q I S L D N P D Y Q Q D F F P K E A K P N G I F K G S T A E N A E Y L R V A P Q S S E F I G A **Stop**

**Figure 1.** Possible structure of the truncated protein encoded by the large form of the truncated EGFRvI mRNA from amplicon 3. The first Met of the over-expressed sequence in phase with the ORF of EGFR is in the intron 10 (bold blue). Twenty-one amino acids are coded (highlighted in blue) before the beginning of exon 11. The Gly (highlighted in red), in position 403 of the reference sequence NM_005228.3, is encoded by a G from intron 10 and 2 G from exon 11. The complete truncated protein contains 829 amino acids. Red: stop codons; Blue: methionines

L P C L Stop E P R N H T R Q D Q A T G P D N C I Q C A H Y I D G P H C V K T C P A G V **Met** G E N N T L V W K Y A D A G H V C H L C H P N C T Y G C T G P G L E G C P T N G P K I P S I A T G Met V G A L L L L L V V A L G I G L F Met R R R H I V R K R T L R R L L Q E R E L V E P L T P S G E A P N Q A L L R I L K E T E F K K I K V L G S G A F G T V Y K G L W I P E G E K V K I P V A I K E L R E A T S P K A N K E I L D E A Y V Met A S V D N P H V C R L L G I C L T S T V Q L I T Q L Met P F G C L L D Y V R E H K D N I G S Q Y L L N W C V Q I A K G Met N Y L E D R R L V H R D L A A R N V L V K T P Q H V K I T D F G L A K L L G A E E K E Y H A E G G K V P I K W Met A L E S I L H R I Y T H Q S D V W S Y G V T V W E L Met T F G S K P Y D G I P A S E I S S I L E K G E R L P Q P P I C T I D V Y Met I Met V K C W Met I D A D S R P K F R E L I I E F S K Met A R D P Q R Y L V I Q G D E R Met H L P S P T D S N F Y R A L Met D E E D Met D D V V D A D E Y L I P Q Q G F F S S P S T S R T P L L S S L S A T S N N S T V A C I D R N G L Q S C P I K E D S F L Q R Y S S D P T G A L T E D S I D D T F L P V P E Y I N Q S V P K R P A G S V Q N P V Y H N Q P L N P A P S R D P H Y Q D P H S T A V G N P E Y L N T V Q P T C V N S T F D S P A H W A Q K G S H Q I S L D N P D Y Q Q D F F P K E A K P N G I F K G S T A E N A E Y L R V A P Q S S E F I G A **Stop**

**Figure 2.** Possible structure of the truncated protein encoded by the small form of the truncated EGFRvI mRNA from amplicon 3. The junction between exons 11 and 15 is highlighted in blue. The first Met of the over-expressed sequence is the amino acid 576 located in exon 15 of the reference sequence NM_005228.3 (bold blue). The complete truncated protein contains 611 amino acids. Red: stop codons; Blue: methionines
