# Supplementary material for: Relationships Linking Amplification Level to Gene Over-Expression in Gliomas
Source: PLoS One. 2010 Dec 8;5(12):e14249. doi: 10.1371/journal.pone.0014249 (PMC2999539; doi:10.1371/journal.pone.0014249)
Supplement: Data S6 — Structure of the amplified EGFR mRNA published by Wong et al. (0.05 MB DOC) [file pone.0014249.s006.doc]

**Supplementary Information data S6**

**Structure of the amplified EGFR mRNA published by Wong et al.**

In their study on amplified and truncated forms of EGFR in gliomas, Wong et al. published the sequence of an mRNA corresponding to the fusion of a truncated EGFR mRNA and a sequence whose origin was not established at that time [1]. The sequence published by Wong et al. (Figure 1) was compared with the human reference genome sequence (http//genome.ucsc.educ). The processed exons located in 5' of the ZNF713 gene (Figures 2 and 3) were fused with exon 14 of the EGFR gene. Both genes are located at 7p11. At fusion, a 5 bp sequence found in the normal counterpart of the ZNF713 exon 1 and EGFR intron 13 was maintained as a single copy. This structure strongly suggests that the junction was formed by a microhomology-based NHEJ mechanism between exon 1 of ZNF713 and intron 13 of EGFR with the formation of an extrachromosomal circular DNA molecule (Figure 3).

The processing of the exons in 5' of the ZNF713 gene suggests that the transcription of the fused mRNA has taken advantage of the formation of the long variant of the ZNF713 mRNA (Figure 2). However, no fusion protein was possible because of the lack of a common open reading frame (not shown). Nevertheless, the synthesis of the receptor may start at ATG in the EGFR sequence (Figure 1). Thus, the over-expression of the EGFRvI variant forms could be associated with an activation of the truncated gene after extrachromosome circularisation of an amplified segment.

...gccagcgtcttgcgccgcgattgcgggaggctgtcctcagagcaggtctggcgcccggtggctccaccggccccaggagcccagtcaccgggcgtcattggctcaggctgcggggccctcggcaccttctcccgccccggggttcccacgcggcgggcggcggcggcggcggcggcgtcaggggcggagcctgccgaagcgccctttgtctgcggaggtcaacatacctggcctaaggaggcagattgagtgactctcactcaccactggtgttgctctttgaaagtggcgcttggcaccagcatgaactccccatcctcagcaatcccatcaggtgttttgggtcttcaacctaaaattctatcttacaagatccttgccaggatgcagatttgaatactatagtgaagtctgtacatgaagaaatgatgcttttagggaggaaaaaaaaaggtaataacaaccttcaagagccccttcatctcaactcggcataaacaaggcaagattctgagagtggccgcccctggaagcagaaattattcttgtggctatccattggctcctgaggctctaatcagagatggggcacctttagtaccaggggagtgactgttgcccataaggtactggacatcaactttcaagagcagccccagctccttaagctgctggtcctggtgcatctgctgactttcatgtagaagatagcagagctttggcgacattacaacataagaactgcagagaggtgtaatcccagtggaagactgaatcgagagactcaaaaaggaag*ttatgccttctcagaatgctgttttttctcaggaggggaacatggaggaggaagaaatgaatgatggctcacagatggtgagatctcag*tgagccaagggagtttgtggagaactctgagtgcatacagtgccacccagagtgcctgcctcaggcc**atg**aacatcac...

**Figure 1**. Sequence of the fused mRNA [1]. Black: processed exons from the 5' region of mRNA of the ZNF713 gene. The exon 1 of the ZNF713 gene, but the last 6 bp in 3', (in italics) was fused with the EGFR gene. The exons in 5' of exon 1 are not included in the RefSeq Gene sequence of ZNF713 (see Figure 2). Blue: exon 14 of the EGFR gene, in bold the first ATG of the sequence. Red: microhomologous sequence in ZNF713 exon 1 and EGFR intron 13.


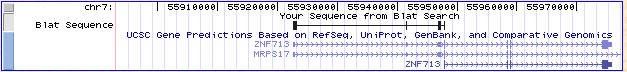


**Figure 2**. Comparison of the sequence with the human reference genome sequence (http//genome.ucsc.educ). Black: sequence published by Wong et al. (without the EGFR region). The processed sequence extended from base 55,922,640 to base 55,947,710.


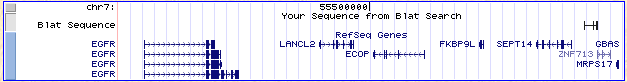


**Figure 3**. Relative positions of EGFR and ZNF713 genes in chromosome 7 (the human reference genome sequence, http//genome.ucsc.educ). Black: sequence published by Wong et al. (without the EGFR region). A fusion between breaks located in the first exon of the reference sequence of ZNF713 and in the intron 13 of EGFR is assumed. The genes located between these two genes were likely included in the amplicon.

Reference

1. Wong AJ, Ruppert JM, Bigner SH, Grzeschik CH, Humphrey PA, et al. (1992) Structural alterations of the epidermal growth factor receptor gene in human gliomas. Proc Natl Acad Sci U S A 89: 2965-2969.
