# Supplementary material for: Relationships Linking Amplification Level to Gene Over-Expression in Gliomas
Source: PLoS One. 2010 Dec 8;5(12):e14249. doi: 10.1371/journal.pone.0014249 (PMC2999539; doi:10.1371/journal.pone.0014249)
Supplement: Data S7 — Telomerase expression. (0.35 MB DOC) [file pone.0014249.s007.doc]

**Supplementary Information data S7**

The telomerase TERT gene was over-expressed about 50-fold in tumour 26. However, the RNA component of the telomerase (TERC), located at 3q26, was not amplified and its expression, measured by RT-Q-PCR (Pelosi, et al. 2007), was poorly expressed in this tumour as compared with the tumours of the control set (Fig 1). In fact, no increase of TERT activity, measured by real-time telomeric repeat amplification (Wege, et al. 2003), was observed in tumour 26 as compared with tumours without amplification of the gene (Fig 2). No deletion, insertion or alternative splicing of the exons was detected by sequencing the whole coding sequence of the mRNA which encodes the normal isoform 1 of the protein (not shown). The level of expression of the protein could not be evaluated as none of the currently available antibodies allowed efficient detection by western blot ((Wu, et al. 2006), unpublished data and E. Blackburn, unpublished data, personal communication). Finally, the telomeres were shorter in tumour 26 (less than 5 kb) than in two gliomas without TERT amplification, which displayed normal telomere lengths of 5 - 15 kb (Figure 3). Thus, despite a high level of mRNA, a high telomerase activity of TERT was not observed in the tumour.

**RT-Q-PCR analysis of RNA component of telomerase (TERC)**

**
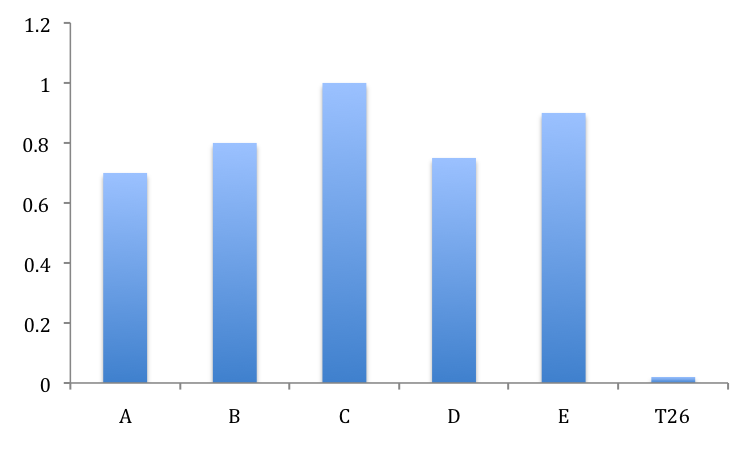
**

The expression of the RNA component of the telomerase (TERC) was measured by RT-Q-PCR as described (Pelosi, et al. 2007). Expression of the 5 tumours of the reference set and of tumour 26 are presented. Relative expressions were normalised using the tumour with the higher level of expression (tumour D). TERC expression was very low in tumour 26.

**Real-time telomeric repeat amplification measurement of the TERT activity**


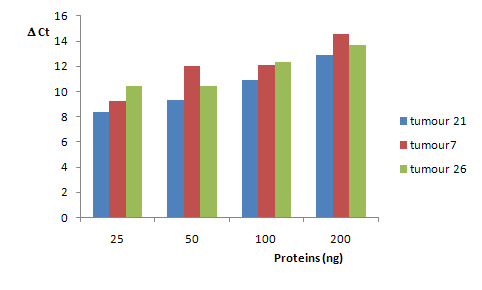


Telomerase activity was measured after extraction of the proteins in CHAPS buffer (TRAPeze CHAPS Lysis buffer, Chemicon) for tumour 26 and two tumours without TERT amplification (tumours 7 and 21). Q-PCR experiments were performed as described (Wege, et al. 2003) using 25, 50, 100 and 200 ng of protein by assay. For negative controls, proteins were denatured for 10 min at 85°C. DeltaCt between negative controls and samples are presented. No significant differences in activity were observed between the 3 tumours

**Telomeric restriction fragment analysis of telomere length**


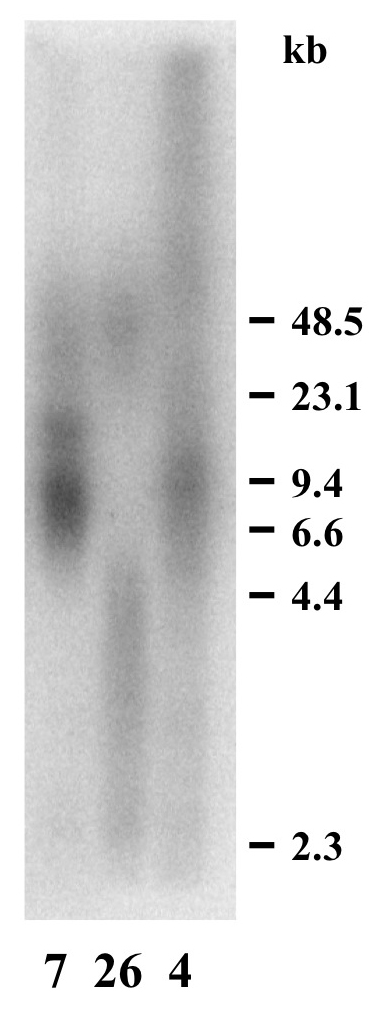


Telomere length was determined by Southern blot (Zhdanova, et al. 2007). Telomeres were shorter in tumour 26 (less than 5 kb) than in the two other gliomas (7 and 4) without TERT amplification, with telomere lengths of 5 - 15 kb. The smears observed in the 25 - 50 kb range likely correspond to contamination of the human DNA by mouse DNA in the preparation from the xenografted tumours.

**References**

Pelosi G, Del Curto B, Trubia M, Nicholson AG, Manzotti M, Veronesi G, Spaggiari L, Maisonneuve P, Pasini F, Terzi A and others. 2007. 3q26 Amplification and polysomy of chromosome 3 in squamous cell lesions of the lung: a fluorescence in situ hybridization study. Clin Cancer Res 13(7):1995-2004.

Wege H, Chui MS, Le HT, Tran JM, Zern MA. 2003. SYBR Green real-time telomeric repeat amplification protocol for the rapid quantification of telomerase activity. Nucleic Acids Res 31(2):E3-3.

Wu YL, Dudognon C, Nguyen E, Hillion J, Pendino F, Tarkanyi I, Aradi J, Lanotte M, Tong JH, Chen GQ and others. 2006. Immunodetection of human telomerase reverse-transcriptase (hTERT) re-appraised: nucleolin and telomerase cross paths. J Cell Sci 119(Pt 13):2797-806.

Zhdanova NS, Minina JM, Karamisheva TV, Draskovic I, Rubtsov NB, Londono-Vallejo JA. 2007. The very long telomeres in Sorex granarius (Soricidae, Eulipothyphla) contain ribosomal DNA. Chromosome Res 15(7):881-90.
