# Supplementary material for: Relationships Linking Amplification Level to Gene Over-Expression in Gliomas
Source: PLoS One. 2010 Dec 8;5(12):e14249. doi: 10.1371/journal.pone.0014249 (PMC2999539; doi:10.1371/journal.pone.0014249)
Supplement: Figure S1 — Structure of the 5 amplicons present in tumour 26. (0.49 MB DOC) [file pone.0014249.s008.doc]

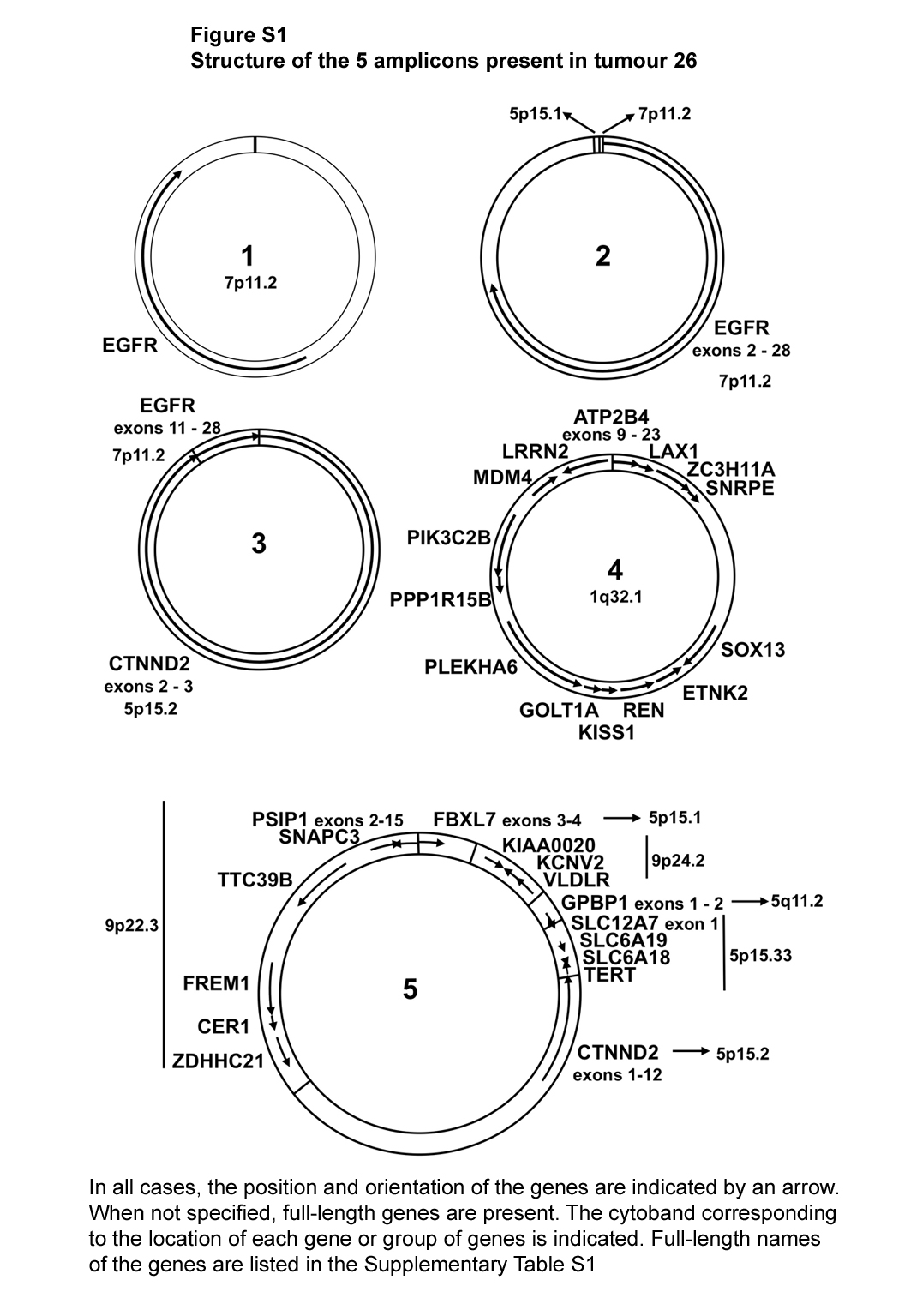


Structure of the amplicons.

Amplicon 1, amplified about 8-fold, corresponds to the circularisation of a single 406 kb-long DNA fragment containing the full-length EGFR gene. Amplicon 2, amplified about 8-fold, contains a 182 kb-long DNA fragment from 7p11.2 associated with two small fragments of 84 and 292 bp, originating respectively from 5p15.1 and 7p11.2. In this amplicon the region containing the first exon of the EGFR gene was missing. Amplicon 3, amplified some 250-fold, contains a 52 kb-long fragment from 7p11.2, corresponding to exons 11 to 28 of the EGFR gene, associated with a fragment of 375 kb from 5p15.2. Amplicon 4 resulted from the circularisation of a 1-Mb fragment from 1q32.1. The 4.6 Mb amplicon 5 corresponded to the association of 6 fragments, ranging from 0.14 to 2.28 Mb. Four of them came from different regions of chromosome 5 (5q11.2, 5p15.1, 5p15.2 and 5p15.33) and the last two fragments from chromosome 9 (9p22.3 and 9p24.2).
