# Supplementary material for: Relationships Linking Amplification Level to Gene Over-Expression in Gliomas
Source: PLoS One. 2010 Dec 8;5(12):e14249. doi: 10.1371/journal.pone.0014249 (PMC2999539; doi:10.1371/journal.pone.0014249)
Supplement: Table S1 — List of the genes amplified in the analysed tumour. Red, overexpressed genes. Blue, genes not over-expressed. Black, not analysed: pseudogenes or truncated genes with a few amplified exons. (0.04 MB DOC) [file pone.0014249.s009.doc]

#### Table S1

**List of the genes amplified in the analysed tumours**

**Tumour 4**

- SEC61G protein transport protein Sec61 subunit gamma. - EGFR epidermal growth factor receptor

- LANCL2 lanC-like protein 2

- VOPP1 vesicular, overexpressed in cancer, prosurvival protein 1.

- SEPT14 septin 14

- ZNF713 zinc finger protein 713

- MRPS17 mitochondrial ribosomal protein S17

- GBAS glioblastoma amplified sequence

- PSPH phosphoserine phosphatase.

- CCT6A chaperonin containing TPC1 subunit 6A isoform

- SUMF2 sulphatase modifying factor 2 precursor.

- PHKG1 phosphorlyase kinase gamma 1.

- CHCHD2 coiled-coil-helix-coiled-coil-helix domain containing protein

**Tumour 7**

- VSTM2A V-set and transmembrane domain containing 2A

- SEC61G protein transport protein Sec61 subunit gamma. - EGFR epidermal growth factor receptor

- LANCL2 lanC-like protein 2

- VOPP1 vesicular, overexpressed in cancer, prosurvival protein 1.

- SEPT14 septin 14

**Tumour 21**

- VSTM2A V-set and transmembrane domain containing 2A

- SEC61G protein transport protein Sec61 subunit gamma. - EGFR epidermal growth factor receptor

**Tumour 22**

- VSTM2A V-set and transmembrane domain containing 2A

- SEC61G protein transport protein Sec61 subunit gamma. - EGFR epidermal growth factor receptor

**Tumour 26**

**Amplicon 1 (x8)**

7p11.2 (0.4 Mb)

- EGFR : epidermal growth factor receptor

**Amplicon 2 (x8)**

a: 7p11.2 (0.2 Mb)

- EGFR : epidermal growth factor receptor. First exon not amplified

b: 5p15.1 (84 bp)

- no gene

# c: 7p11.2 (292 bp)

# - no gene

# Amplicon 3 (x250)

a: 7p11.2 (0.4 Mb)

- EGFR : epidermal growth factor receptor. Exons 11 to 28

b: 5p15.2 (52Kb)

- CTNND2 : catenin (cadherin-associated protein) delta 2. Exons 2 and 3

**Amplicon 4. (x10)**

1q32.1 (1Mb )

- ATP2B4 : ATPase, Ca++ transporting, plasma membrane 4. Exons 9 to 23

- LAX1 : membrane-associated adaptor protein LAX.

- ZC3H11A : zinc finger CCCH-type containing 11A (pseudo gene)

- SNRPE : small nuclear ribonucleoprotein polypeptide E (pseudo gene)

- SOX13 : SRY (sex-determining region Y)-box 13

- ETNK2 : ethanolamine kinase 2

- REN : kidney mRNA fragment for renin

- KISS1 : KiSS-1 metastasis-suppressor

- GOLT1A : Golgi transport 1 homologue A

- PLEKHA6 : phosphoinositol 3-phosphate-binding protein-3

- PPP1R15B : protein phosphatase 1, regulatory (inhibitor) subunit 15B

- PIK3C2B : phosphoinositide-3-kinase, class 2, beta polypeptide

- MDM4 : transformed 3T3 cell dmin4, p53 binding protein

- LRRN2 : leucine-rich repeat neuronal 2. Formerly GAC1

**Amplicon 5 (x15)**

# a: 5p15.2 (2.28Mb)

# - CTNND2 : catenin (cadherin-associated protein) delta 2. Exons 1 to 12

# b: 9p22.3 (1 Mb)

# - ZDHHC21 : zinc finger DHHC domain containing 21

- CER1 : Cerberus 1. Cytokine member of the cysteine knot superfamily

- FREM1 : FRAS1 related extracellular matrix 1

- TTC39B : tetratricopeptide repeat domain 39B

- SNAPC3 : small nuclear RNA activating complex

- PSIP1 : PC4 and SFRS1 interacting protein 1. Exons 2 to 15 pseudo gene

c: 5p15.1 (420 Kb)

- FBXL7 : F-box and leucine-rich repeat protein 7. Exons 3 and 4

d: 9p24.2 (464 Kb )

- VLDLR : very-low-density lipoprotein receptor

- KCNV2 : potassium channel subfamily V member 2

- KIAA0020 : minor histocompatibility antigen HA-8

e: 5q11.2 (143 Kb)

- GPBP1 : vasculin, expressed in atherogenesis. Exons 1 and 2

f: 5p15.33 (197 Kb)

- SLC12A7 : solute carrier family 12 (K/Cl transporters), member 7. Exon 1.

- SLC6A19 : solute carrier family 6 (neurotransmitter transporter), member 19

- SLC6A18 : solute carrier family 6 (neurotransmitter transporter), member 18

- TERT : Telomerase reverse transcriptase

**Tumour 30**

- VSTM2A V-set and transmembrane domain containing 2A

- SEC61G protein transport protein Sec61 subunit gamma. - - EGFR epidermal growth factor receptor

- LANCL2 lanC-like protein 2

- VOPP1 vesicular, overexpressed in cancer, prosurvival protein 1.

Red, overexpressed genes. Blue, genes not over-expressed. Black, not analysed: pseudogenes or truncated genes with a few amplified exons.
